# Supplementary material for: Comparative Assessment of Docking Programs for Docking and Virtual Screening of Ribosomal Oxazolidinone Antibacterial Agents
Source: Antibiotics (Basel). 2023 Feb 24;12(3):463. doi: 10.3390/antibiotics12030463 (PMC10044086; doi:10.3390/antibiotics12030463)
Supplement: Supplementary file 1 [file antibiotics-12-00463-s001.zip › antibiotics-2193858-supplementary.pdf]

antibiotics

MDPI

Supplementary File

3CPW	3CXC	3DLL
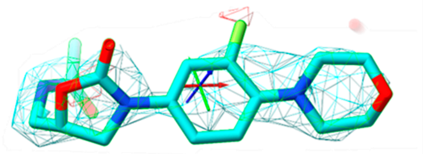	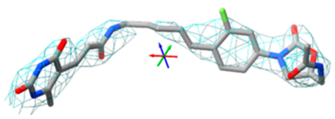	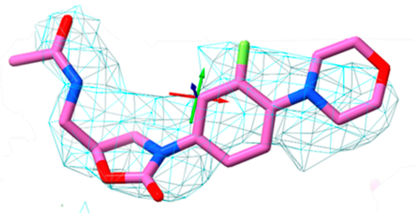
**2.7 Å**	**3.0 Å**	**3.5 Å**
Haloarcula marismortui	***Haloarcula marismortui***	***Deinococcus radiodurans***

4WFA	6DDD	6DDG
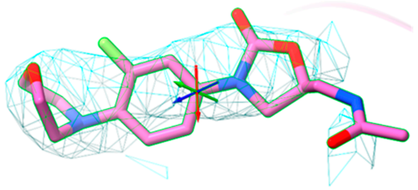	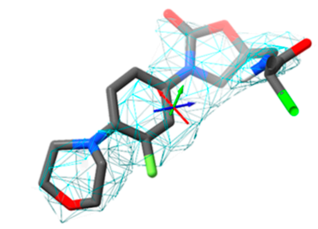	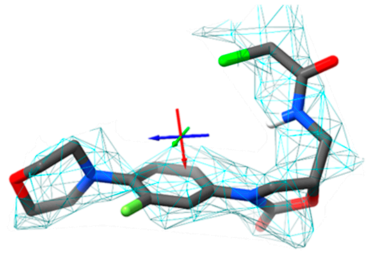
**3.4 Å**	**3.1 Å**	**3.1 Å**
Staphylococcus aureus	***Methicillin resistant Staphylococcus aureus***	***Methicillin resistant Staphylococcus aureus***

6QUL	6WQN	6WQQ
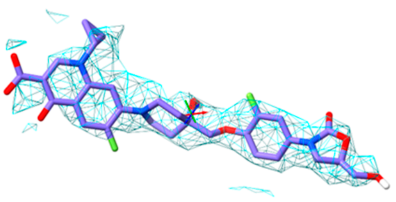	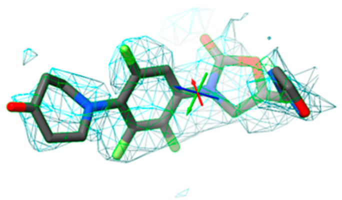	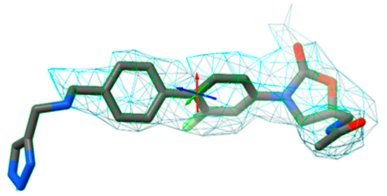
**3.0 Å**	**2.9 Å**	**3.1 Å**
Escherichia coli	***Methicillin resistant Staphylococcus aureus***	***Methicillin resistant Staphylococcus aureus***

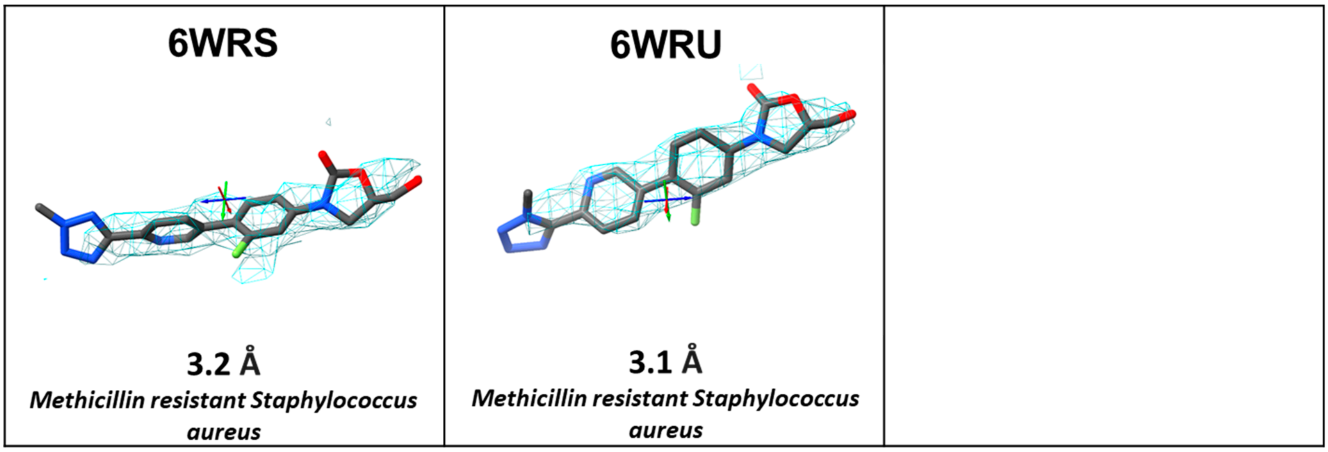

Figure S1. Electron dentistry maps (at binding site) for selected ribosomal crystal structures: 3CPW (2.7 Å), 3CXC (3.0 Å), 3DLL (3.5 Å), 4WFA (3.4 Å), 6DDD (3.1 Å), 6DDG (3.1 Å), 6QUL (3.0 Å), 6WQN (2.9 Å), 6WQQ (3.1 Å), 6WRS (3.2 Å), and 6WRU (3.1 Å). The resolution is shown in the mesh in blue, with the XYZ axis indicator displayed (red, green, blue, respectively). Species type is also listed. Note: the binding site residues are not shown for clarity of the electron density, due to other residue that obstructed the full view of the ligand. ChimeraX (v.1.4) was used for the visualization, in conjunction with the ISOLDE toolkit.

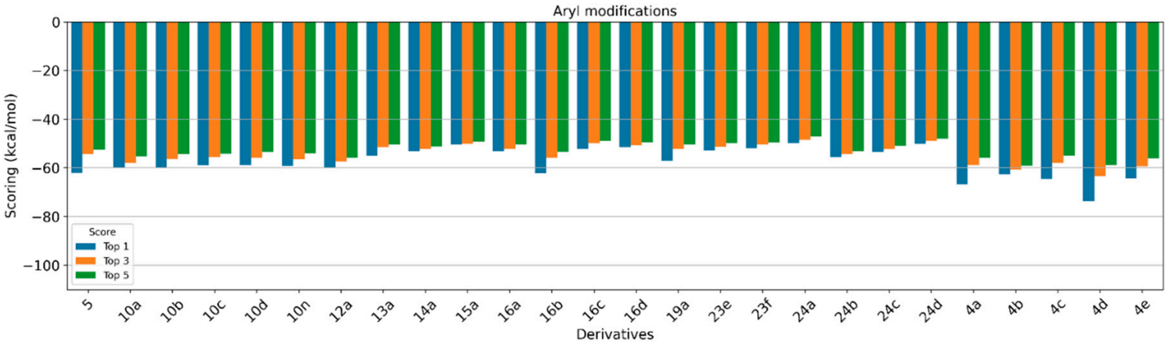
Aryl modifications
Scoring (kcal/mol)
Derivatives
Score
Top 1
Top 3
Top 5
5 10a 10b 10c 10d 10n 12a 13a 14a 15a 16a 16b 16c 16d 19a 23e 23f 24a 24b 24c 24d 4a 4b 4c 4d 4e

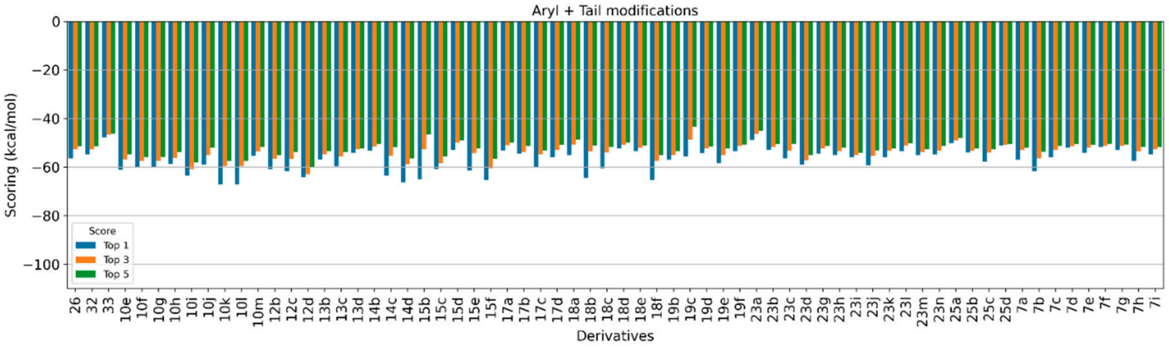
Aryl + Tail modifications
Scoring (kcal/mol)
Derivatives
Score
Top 1
Top 3
Top 5
26 32 33 10e 10f 10g 10h 10i 10j 10k 10l 10m 12b 12c 12d 13b 13c 13d 14b 14c 14d 15b 15c 15d 15e 15f 17a 17b 17c 17d 18a 18b 18c 18d 18e 18f 19b 19c 19d 19e 19f 23a 23b 23c 23d 23g 23h 23i 23j 23k 23l 23m 23n 25a 25b 25c 25d 7a 7b 7c 7d 7e 7f 7g 7h 7i

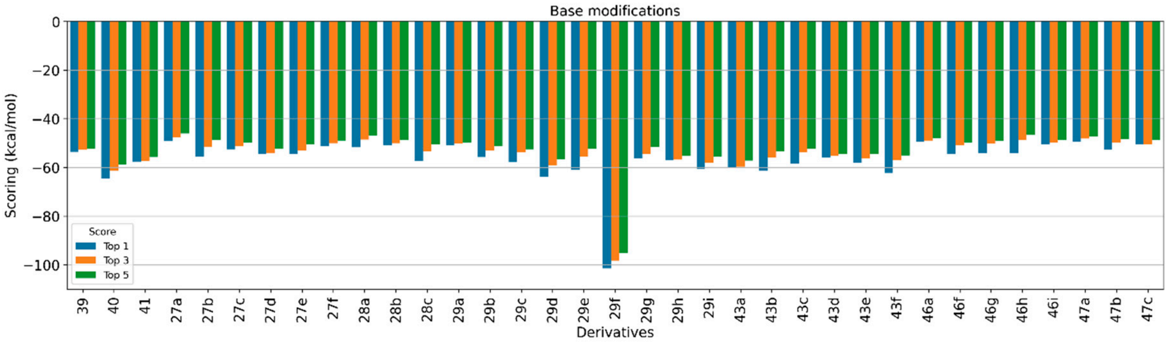
Base modifications
Scoring (kcal/mol)
Derivatives
Score
Top 1
Top 3
Top 5
39 40 41 27a 27b 27c 27d 27e 27f 28a 28b 28c 29a 29b 29c 29d 29e 29f 29g 29h 29i 43a 43b 43c 43d 43e 43f 46a 46f 46g 46h 46i 47a 47b 47c

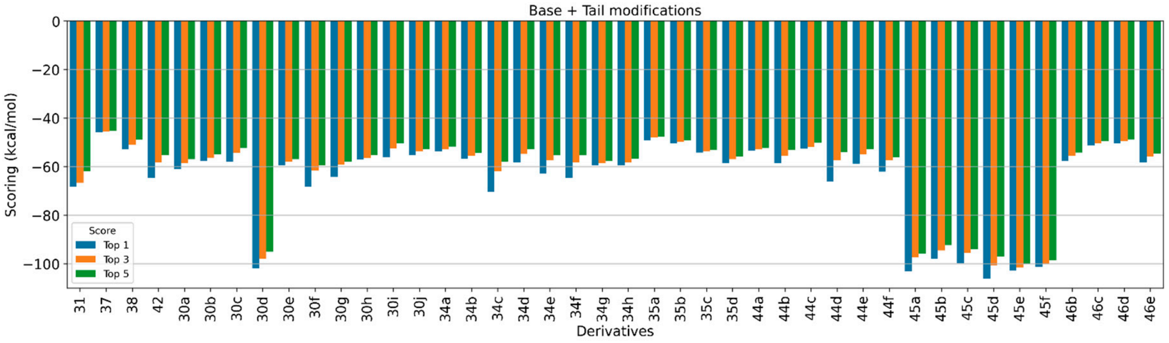
Base + Tail modifications
Scoring (kcal/mol)
Derivatives
Score
Top 1
Top 3
Top 5
31 37 38 42 30a 30b 30c 30d 30e 30f 30g 30h 30i 30j 34a 34b 34c 34d 34e 34f 34g 34h 35a 35b 35c 35d 44a 44b 44c 44d 44e 44f 45a 45b 45c 45d 45e 45f 46b 46c 46d 46e

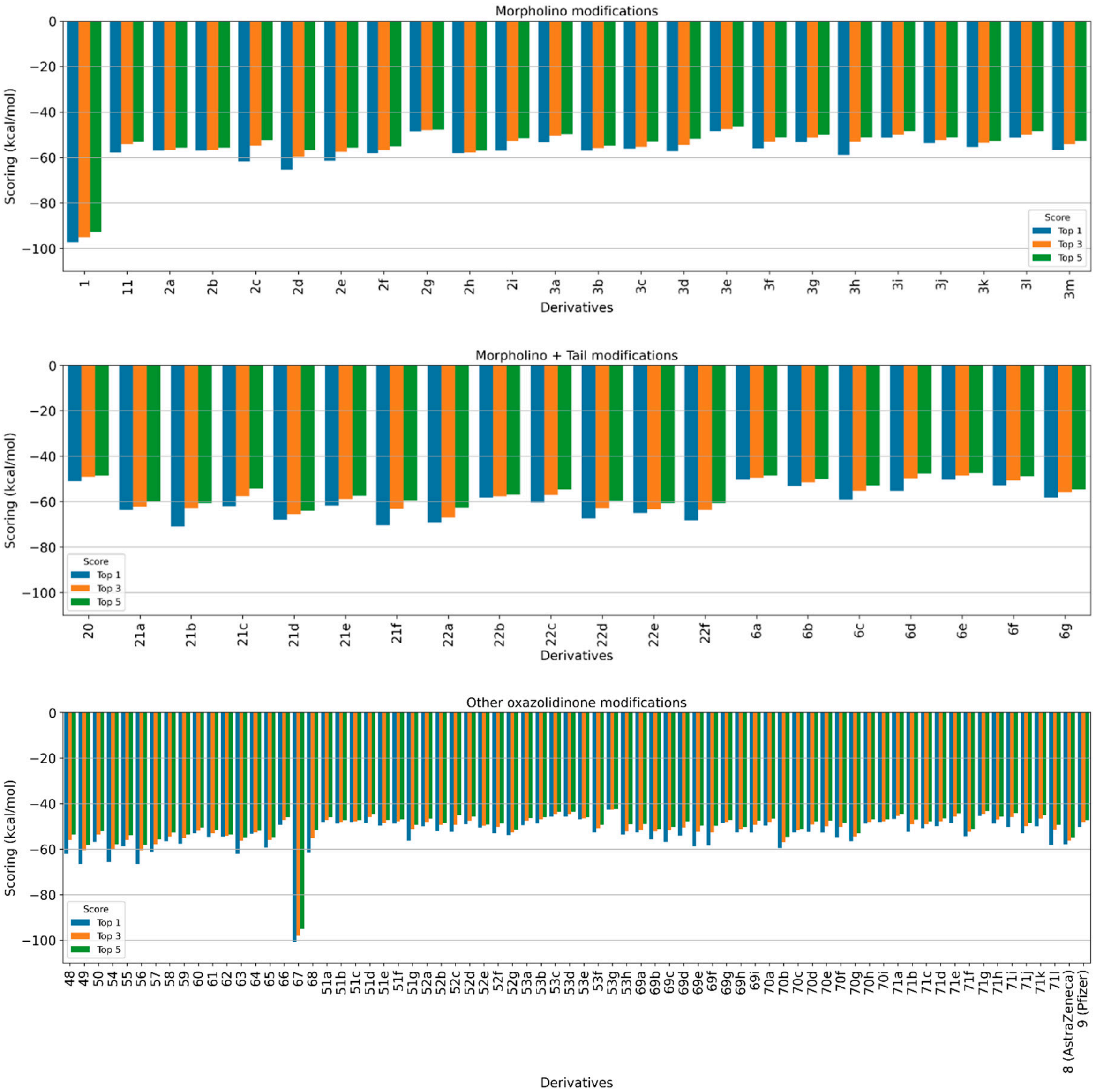

Figure S2. DOCK 6 scoring of the dataset split into the specific structural modification groups. Many values stayed above -60kcal/mol, while the top performers were all below -90kcal/mol.

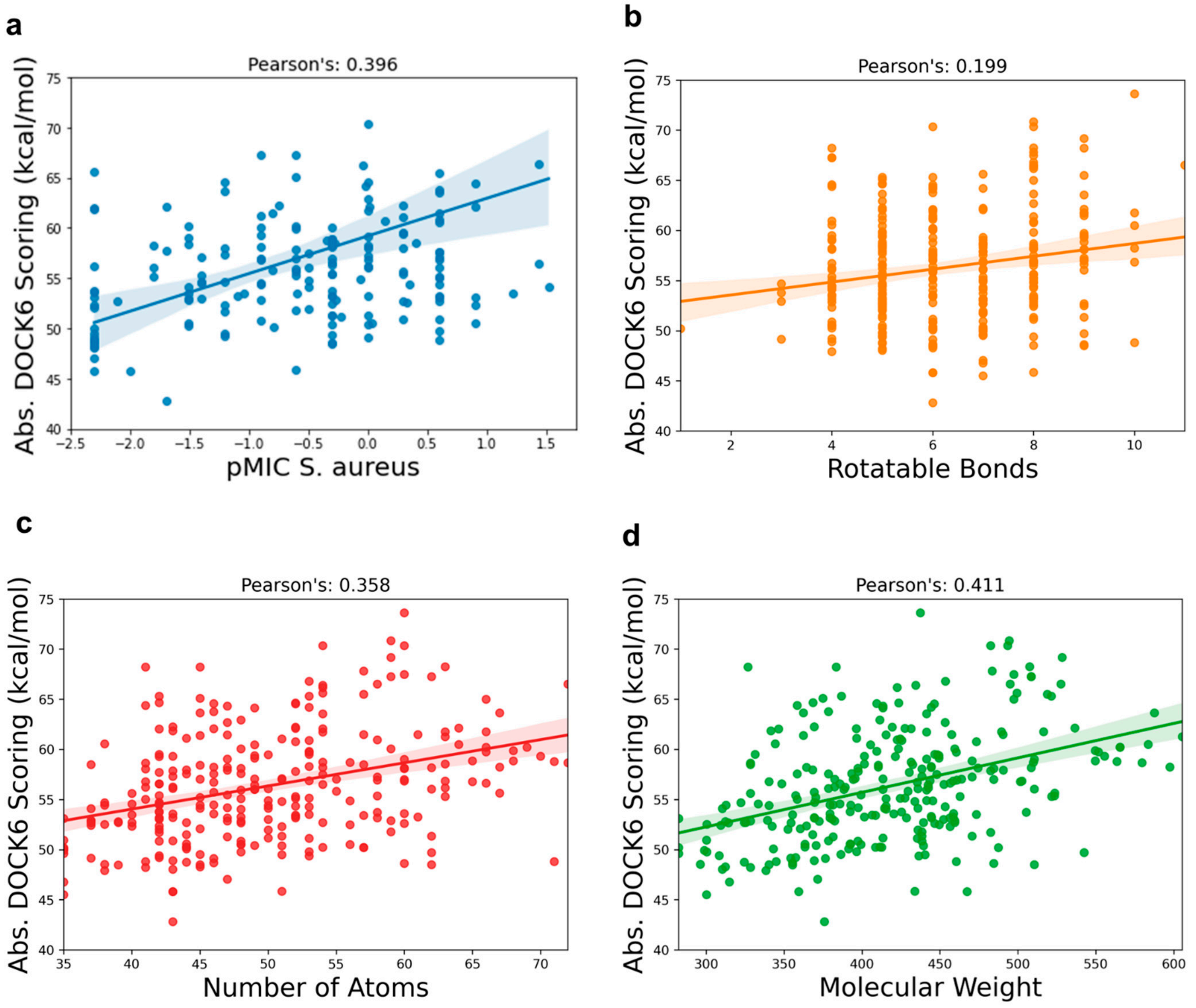

Figure S3. Distribution of molecular features of the derivatives (**a**) pMIC of *S. aureus* docked against their respective PDBs (4WFA), and the (**b**) number of rotatable bonds, (**c**) molecular weight, and the (**d**) number of atoms in the molecule vs the absolute docking score for 4WFA. Pearson values are displayed.

	Top 1	Top 2	Top 3
1	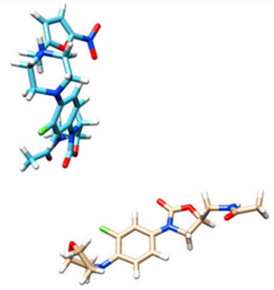	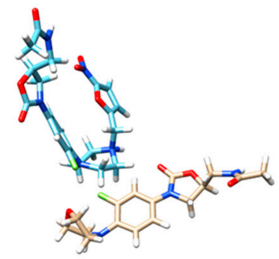	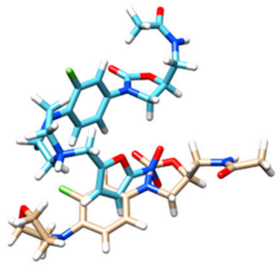
3l	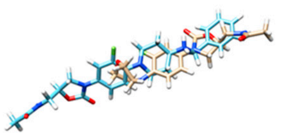	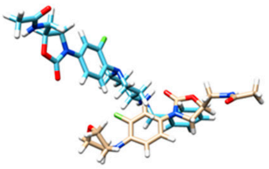	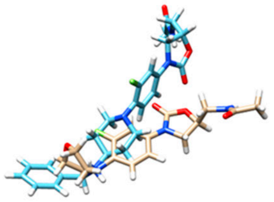
29f	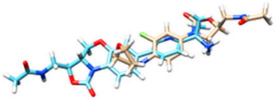	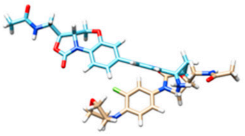	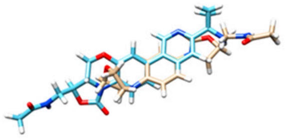
30d	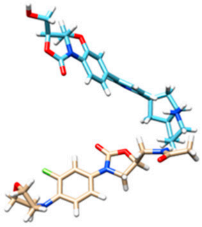	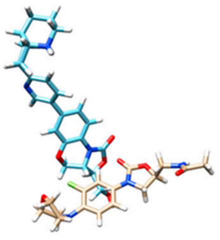	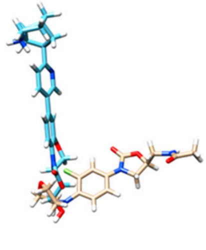
45a	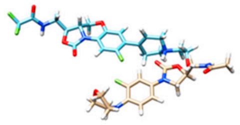	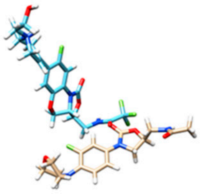	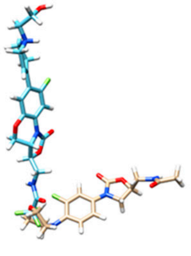
45b	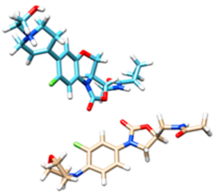	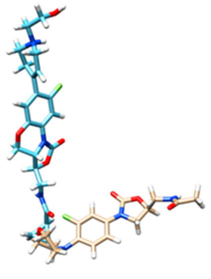	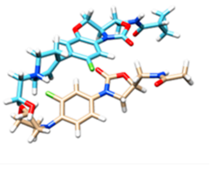

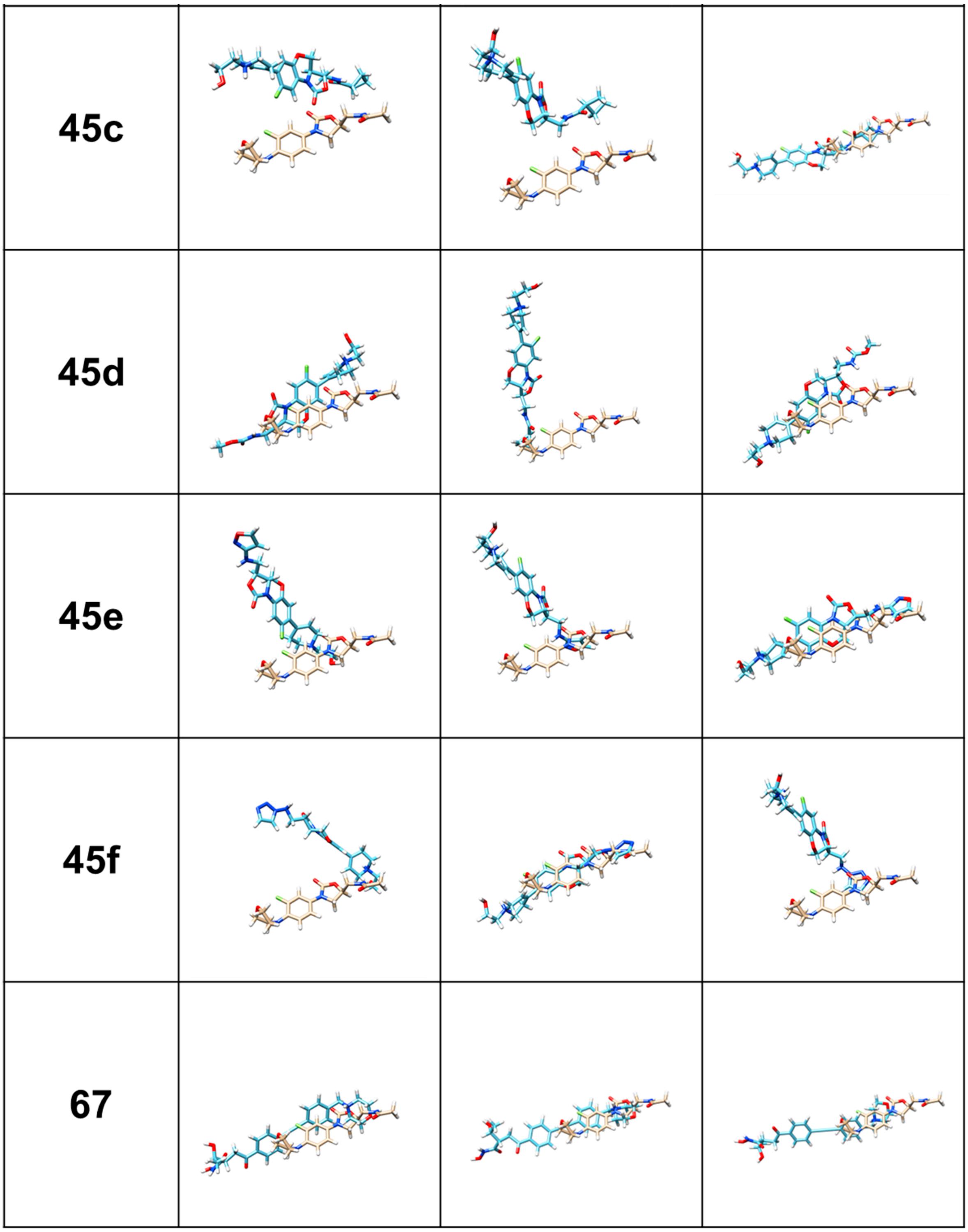

Figure S4. DOCK 6 Top three poses (blue) of each top-performing derivative superimposed against linezolid (beige) in the pocket of 4WFA. Note: the nucleotide residues in the binding site are not shown for clarity of the posing.

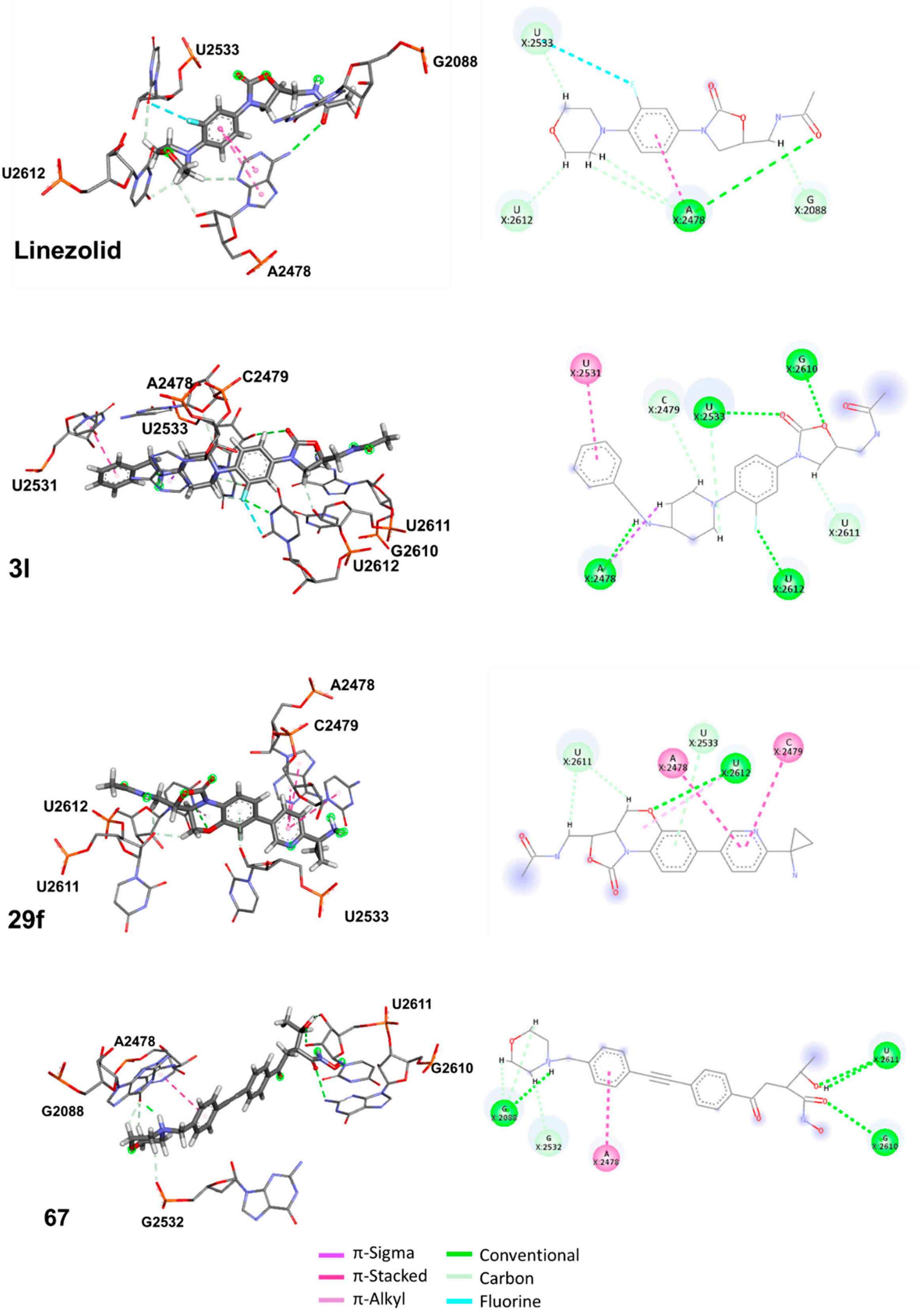

Figure S5. The intermolecular interactions of linezolid and derivatives 3l, 29f, and 67 with the active site pockets of *S. aureus*. Left side (3D representation): the docked residues of the target are shown in the rose stick model (labelled), and compounds are in the grey stick. Right side (2D representation): compounds shown in grey with the residue interactions displayed. *E. coli* residue numbering

is used for both 3D and 2D representations. The dashed lines represent the different interactions, and their colour is the interaction type. The coloured circles represent residues with letter code (A for Adenine, C for Cytosine, G for Guanine, or U for Uracil), chain identifier (Chain X) and residue number. 2D visualization was carried out using BIOVIA Discovery Studio.

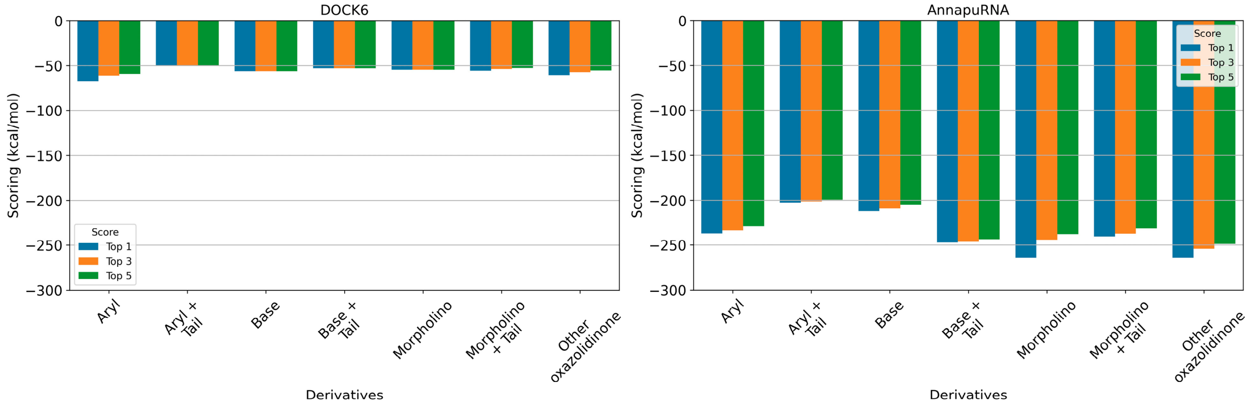

Figure 6. DOCK 6 scoring compared to the re-scored values from AnnapuRNA.

Table S1. Docking score and descriptors of training set compounds.

ID	DOCK 6 score (kcal/mol)	MolWt[A]	TPSA[B]	nRotB[C]	HBD[D]	HBA[E]	LogP	Num-Rings[F]	*S. aureus* pMIC
9	-47.36	487.15	143.31	1	2	9	1.20	6	-0.80
10e	-44.70	591.14	146.38	4	3	8	1.57	6	-0.90
13b	-51.01	443.14	97.38	5	3	7	0.37	3	0.60
14c	-56.02	441.15	106.61	5	3	8	-0.18	3	0.60
16c	-51.09	456.09	84.00	4	1	7	3.44	5	-1.20
18e	-51.00	355.10	95.52	3	2	5	2.05	4	1.22
19a	-51.44	394.17	102.42	5	2	6	-0.07	3	-0.90
19f	-54.26	406.16	97.56	5	1	6	3.42	4	-1.51
23e	-50.77	372.16	84.42	4	1	5	2.67	3	-0.90
23g	-50.35	392.11	84.42	4	1	5	3.48	3	-0.90
23m	-54.19	424.15	97.56	5	1	6	3.56	4	-1.81
2a	-54.38	458.22	121.32	6	2	9	0.76	4	-0.90
2b	-50.40	502.21	138.44	8	1	10	1.51	4	0.00
2f	-47.96	518.15	120.62	7	1	8	3.00	4	-0.90
3e	-44.66	510.19	83.14	7	2	6	4.27	4	-0.30
3i	-56.92	510.19	83.14	7	2	6	4.27	4	-0.30
3j	-49.98	504.12	73.91	6	2	5	4.13	4	-0.30
43b	-50.01	457.20	88.18	4	1	5	2.85	5	-0.81
43d	-55.06	429.17	88.18	4	1	5	2.07	5	-0.80
43f	-53.28	419.15	108.41	4	2	6	0.66	4	-0.74
44d	-58.15	421.16	100.57	5	2	7	1.35	4	-0.04
45c	-93.99	421.15	71.11	4	1	6	2.38	5	1.52
46i	-47.38	399.14	71.11	3	1	5	2.28	4	0.04
51b	-47.42	324.15	67.43	4	2	3	3.29	3	-2.30
51c	-46.80	328.12	67.43	4	2	3	3.12	3	-2.30
52d	-45.67	376.11	93.73	6	2	5	2.54	3	-2.30
52f	-53.89	380.06	84.50	5	2	4	3.18	3	-2.30
53e	-44.17	371.13	71.62	5	3	4	3.55	3	-2.30
6d	-53.47	462.10	89.75	7	0	5	5.72	4	-1.40
7b	-51.63	341.12	62.39	4	3	3	3.54	3	-2.30

[A] Molecular weight of the derivative

[B] Topological Polar Surface Area of the derivative

[C] Number of rotatable bonds in the derivative

[D] Number of H bond donors in the derivative

[E] Number of H bond acceptors in the derivative

[F] Number of rings in the derivative

Table S2. Experimental and predicted pMIC values of the rescores for the test set compounds with descriptors.

ID	DOCK 6 score (kcal/mol)	MolWt[A]	TPSA[B]	nRotB[C]	HBD[D]	HBA[E]	LogP	Num-Rings[F]	*S. aureus* pMIC	Predicted pMIC
1	-97.35	461.17	121.40	7	1	8	2.11	4	0.00	0.80
8	-57.94	489.02	71.11	4	1	5	3.35	3	0.00	0.85
11	-57.95	368.13	75.94	4	1	5	2.60	4	0.30	-0.72
20	-50.88	378.12	77.55	4	0	7	2.76	5	0.60	-0.55
26	-56.51	452.16	123.41	6	3	9	0.54	4	1.44	-0.37
32	-54.69	322.11	98.48	2	2	5	1.27	4	-1.20	-1.43
37	-45.86	433.11	66.84	6	1	4	5.52	4	-0.60	-1.86
38	-52.94	339.07	86.45	2	1	6	2.42	4	-1.51	-0.87
39	-53.56	379.13	82.43	3	1	4	2.75	4	-0.59	-1.07
40	-64.63	423.15	113.10	4	2	7	1.96	5	-1.20	-0.76
41	-57.60	393.13	100.99	3	2	6	1.31	4	0.60	-0.53
48	-62.13	535.14	62.21	8	0	4	6.47	5	0.90	-0.70
54	-65.64	498.09	92.79	6	2	6	4.64	5	-2.30	-0.84
59	-57.71	429.01	63.25	5	2	5	4.97	4	-1.70	-0.93
61	-54.72	369.09	63.25	5	2	5	4.34	4	-1.40	-1.52
62	-54.55	385.07	63.25	5	2	5	4.86	4	-1.40	-1.52
63	-62.11	427.14	63.25	6	2	5	5.87	5	-1.70	-1.60
64	-53.26	401.12	63.25	5	2	5	5.36	5	-1.10	-1.86
10b	-59.88	549.20	137.15	4	3	7	2.22	6	-0.60	-0.82
10c	-58.86	549.20	137.15	4	3	7	2.22	6	-0.30	-0.86
10d	-58.79	563.22	137.15	5	3	7	2.61	6	-0.30	-1.04
10f	-60.23	565.20	146.38	4	3	8	2.44	6	-1.51	-0.74
10g	-60.23	551.18	146.38	4	3	8	2.05	6	-0.60	-0.76
10h	-58.69	579.21	146.38	5	3	8	2.83	6	-0.30	-0.98
10i	-63.64	587.15	163.45	5	3	9	0.48	6	-1.20	-0.11
10j	-59.01	510.16	137.51	3	3	8	0.92	6	-1.51	-0.40
10k	-67.28	508.18	128.28	3	3	7	2.08	6	-0.90	-0.49
10l	-67.28	494.16	128.28	3	3	7	1.69	6	-0.60	-0.51
10m	-55.29	507.19	120.08	4	3	6	1.63	6	-0.60	-0.99
10n	-59.33	555.20	123.71	6	1	9	2.05	5	-0.90	0.55
12b	-60.90	425.15	97.38	5	3	7	0.23	3	0.60	0.39
12d	-64.20	444.16	122.05	5	2	9	0.36	5	-0.02	0.08
13a	-55.14	427.17	114.45	5	3	7	-0.43	3	0.60	-0.09

[A] Molecular weight of the derivative

[B] Topological Polar Surface Area of the derivative

[C] Number of rotatable bonds in the derivative

[D] Number of H bond donors in the derivative

[E] Number of H bond acceptors in the derivative

[F] Number of rings in the derivative

14a	-53.17	428.15	111.65	5	2	7	0.00	3	0.60	0.00
14d	-66.39	434.17	123.41	6	3	9	0.40	4	1.44	-0.15
15a	-50.50	392.19	58.64	4	1	3	4.18	3	-1.51	-0.98
15b	-65.10	374.11	84.42	4	1	5	3.34	3	-0.60	-0.63
15c	-60.77	473.12	108.41	4	2	6	1.20	4	0.14	0.14
15d	-52.88	459.18	108.41	5	2	6	1.44	5	-0.27	-0.89
16a	-53.20	369.12	88.83	4	1	6	2.00	4	0.60	-0.83
16b	-62.24	369.12	88.83	4	1	6	2.00	4	0.30	-0.48
17a	-53.16	379.12	90.44	4	0	8	2.15	5	0.90	-0.38
17b	-54.46	379.12	90.44	4	0	8	2.15	5	0.60	-0.33
17c	-60.06	422.09	84.42	5	1	5	3.31	3	-0.30	-0.45
17d	-56.11	378.12	77.55	4	0	7	2.76	5	0.60	-0.35
18a	-55.02	358.10	88.10	3	2	5	2.14	4	0.60	-1.19
18b	-64.40	358.10	88.10	3	2	5	2.14	4	0.90	-0.83
18c	-60.55	344.08	95.77	3	2	5	2.24	4	0.60	-1.42
18d	-52.34	342.10	78.87	3	2	4	2.30	4	0.90	-1.50
19c	-55.65	523.21	130.61	4	1	8	3.29	5	-0.60	-0.49
19e	-58.30	444.14	117.37	5	2	8	1.62	5	0.00	-0.72
21c	-62.05	346.10	84.50	5	2	4	2.53	3	-2.30	-1.45
23a	-48.90	369.11	95.52	3	2	5	2.36	4	0.60	-1.61
23b	-52.86	382.16	86.03	4	0	7	2.82	4	-1.51	-0.43
23d	-59.12	449.12	121.29	4	0	10	2.62	6	0.00	-0.26
23i	-55.94	440.08	84.42	5	1	5	3.45	3	-0.60	-0.45
23k	-56.01	388.15	93.65	4	1	6	2.89	3	-1.81	-0.69
23n	-54.87	445.16	108.41	5	2	6	1.05	5	-0.06	-0.84
24b	-55.46	399.12	96.97	4	2	5	2.28	4	0.30	-1.22
25d	-51.13	435.14	117.64	4	2	7	0.88	4	-0.23	-0.59
27a	-49.23	357.11	80.76	3	1	5	2.11	4	-1.20	-0.96
27b	-55.40	357.11	80.76	3	1	5	2.11	4	-0.30	-0.72
27c	-52.48	449.16	128.64	5	3	7	0.02	4	-0.30	-0.83
27e	-54.40	407.13	71.11	3	1	6	2.13	5	0.36	0.08
27f	-51.30	381.13	97.83	4	1	6	2.17	4	-0.30	-1.10
28a	-51.72	339.12	80.76	3	1	5	1.97	4	-1.20	-1.02
28b	-50.84	339.12	80.76	3	1	5	1.97	4	-0.90	-1.06
28c	-57.32	364.12	104.55	3	1	6	1.84	4	-1.20	-0.96
29a	-50.86	397.13	107.06	4	1	7	1.76	4	0.30	-0.82
29b	-55.72	369.13	89.99	4	1	6	1.98	4	0.30	-0.76
29c	-57.87	338.13	67.87	3	1	4	2.58	4	-0.60	-0.87
29d	-63.77	378.13	104.55	4	1	6	2.04	4	0.60	-0.82
29e	-61.05	424.14	110.30	4	1	7	1.93	5	0.30	-0.56
29f	-101.44	421.15	124.36	4	1	9	1.16	5	0.60	1.30
29g	-56.27	382.11	104.55	3	1	6	1.98	4	-0.30	-0.84
29h	-57.10	394.16	106.78	4	2	6	1.92	5	0.60	-1.42
2c	-61.76	516.22	138.44	8	1	10	1.90	4	0.00	-0.08

2d	-65.50	474.17	133.76	7	1	9	1.94	4	0.60	-0.35
2e	-61.48	490.14	120.62	7	1	9	2.41	4	0.60	-0.08
2g	-48.62	484.19	120.62	7	1	8	2.35	4	-0.30	-0.87
2h	-58.09	484.19	120.62	7	1	8	2.35	4	-0.30	-0.51
2i	-57.02	484.19	120.62	7	1	8	2.35	4	-0.60	-0.55
30b	-57.57	358.12	112.35	4	3	7	0.85	4	0.60	-1.31
34c	-70.35	482.14	132.14	5	1	10	1.58	6	0.00	0.18
34d	-58.21	364.16	86.03	4	0	7	2.69	4	-1.81	-0.38
34e	-62.94	411.13	102.60	4	1	8	2.32	5	0.00	-0.30
34f	-64.57	409.12	99.44	4	0	8	2.47	5	0.00	0.01
34g	-59.49	448.14	113.08	5	0	10	1.91	6	0.30	0.07
34h	-59.39	449.14	125.97	5	0	11	1.30	6	0.30	0.14
35a	-49.15	354.08	118.54	2	1	6	1.33	4	0.00	-1.44
35b	-50.27	411.10	133.67	3	1	8	1.82	5	-1.51	-1.37
35c	-54.14	414.10	124.29	3	1	7	1.42	5	-1.51	-0.99
35d	-58.43	410.11	125.46	4	1	8	1.10	5	-1.51	-0.74
3a	-53.26	458.21	73.91	7	2	5	3.18	4	-0.30	-0.82
3b	-56.94	460.17	73.91	6	2	5	4.02	4	-0.30	-0.77
3c	-56.16	442.24	73.91	6	2	5	4.00	4	0.00	-1.00
3d	-57.24	504.12	73.91	6	2	5	4.13	4	0.00	-0.28
3f	-56.06	510.19	83.14	7	2	6	4.27	4	0.00	-0.55
3g	-53.18	444.20	73.91	6	2	5	3.51	4	0.00	-0.91
3h	-58.86	470.23	94.14	7	3	6	3.42	4	-0.30	-1.16
3k	-55.37	522.11	73.91	6	2	5	4.27	4	-0.30	-0.19
3l	-51.34	438.15	132.44	6	2	7	1.63	4	0.00	-1.68
3m	-56.62	456.25	73.91	7	2	5	3.68	4	-0.30	-0.90
43a	-60.07	459.18	97.41	4	1	6	1.84	5	-0.90	0.00
43c	-58.56	401.16	108.41	4	2	6	0.52	4	0.41	-0.53
43e	-58.05	340.12	93.65	3	1	6	1.37	4	-1.20	-0.70
44a	-53.50	459.14	91.34	5	2	6	1.67	4	-1.04	-0.14
44b	-58.50	431.19	91.34	6	2	6	1.52	5	-0.28	-0.70
44c	-52.63	445.20	91.34	6	2	6	1.91	5	0.33	-0.91
44e	-58.73	430.17	100.30	6	2	8	2.09	5	-0.35	-0.71
44f	-62.06	430.18	104.98	6	2	9	0.83	5	0.01	-0.01
45b	-97.89	407.13	71.11	4	1	6	1.99	5	1.52	1.61
45d	-106.04	397.11	80.34	3	1	7	1.82	4	1.52	2.31
45e	-102.77	406.11	80.07	4	1	8	2.57	5	1.52	1.78
46a	-49.48	440.12	101.07	4	1	7	2.64	5	-1.20	-0.79
46d	-50.43	370.16	93.65	4	1	6	2.75	3	-0.30	-1.06
46f	-54.46	381.12	71.11	3	1	6	1.60	4	-0.90	0.26
46g	-51.04	377.14	80.34	3	1	6	0.88	5	0.04	-0.23
46h	-54.19	397.11	88.18	3	1	6	0.62	4	1.52	0.27
47a	-49.38	436.17	102.24	4	1	7	2.06	5	-0.30	-0.67
47b	-52.67	354.17	84.42	4	1	5	2.53	3	-2.11	-1.04

47c	-50.53	363.16	71.11	3	1	5	1.04	4	0.90	-0.14
51a	-48.04	355.12	110.57	5	2	5	2.89	3	-2.30	-2.58
51d	-48.51	340.14	76.66	5	2	4	2.99	3	-2.30	-1.97
51f	-48.81	344.09	67.43	4	2	3	3.63	3	-2.30	-1.91
51g	-56.23	388.04	67.43	4	2	3	3.74	3	-2.30	-1.15
52a	-50.07	391.08	127.64	6	2	6	2.44	3	-2.30	-2.39
52b	-52.09	360.11	84.50	5	2	4	2.84	3	-2.30	-1.78
52c	-52.37	364.09	84.50	5	2	4	2.67	3	-2.30	-1.66
52g	-53.76	424.01	84.50	5	2	4	3.29	3	-2.30	-1.14
53a	-49.51	386.10	105.53	5	3	5	3.44	3	-2.30	-2.44
53b	-48.70	355.14	62.39	4	3	3	3.84	3	-2.30	-1.94
53c	-45.83	359.11	62.39	4	3	3	3.68	3	-2.00	-1.94
53d	-45.83	467.02	62.39	4	3	3	4.14	3	-2.30	-0.84
53f	-52.70	459.14	106.61	5	3	8	-0.04	3	0.60	0.53
53g	-42.81	375.08	62.39	4	3	3	4.19	3	-1.70	-2.06
53h	-53.59	419.03	62.39	4	3	3	4.30	3	-2.30	-1.17
6b	-53.18	455.06	71.11	5	1	5	2.79	3	-0.30	0.28
6e	-50.40	439.14	84.25	5	1	6	2.89	4	0.00	-0.50
6f	-52.71	407.09	71.11	4	1	5	2.82	3	0.30	-0.11
6g	-58.20	444.16	91.42	5	1	7	1.77	3	-0.30	0.58
70a	-49.76	399.14	71.11	5	1	5	2.17	3	0.60	-0.28
7a	-57.04	463.12	76.07	7	1	5	5.53	4	-1.40	-1.32
7c	-56.07	435.10	46.61	6	0	3	5.95	4	-0.49	-0.99
7d	-51.96	435.10	46.61	6	0	3	5.95	4	-0.49	-1.15
7e	-54.12	435.10	46.61	6	0	3	5.95	4	-0.49	-1.07
7f	-51.79	438.22	86.03	7	0	7	4.17	4	-0.60	-0.90
7g	-53.02	451.07	46.61	6	0	3	6.47	4	-1.40	-1.12
7h	-57.50	447.12	55.84	7	0	4	5.82	4	-0.49	-0.99
7i	-54.82	433.11	66.84	6	1	4	5.52	4	-0.49	-1.52

Supplementary Discussion 1. Field-template pharmacophore

To expand the molecular docking results, we performed ligand-based reverse pharmacophore modelling of the active compounds of known oxazolidinone-based antibacterial drugs. The approach uses a molecular field points-based similarity method to generate a series of low-energy conformations for each compound. The ligands from the chosen crystal structure (linezolid [39, 40, 41], cadazolid [72], radezolid [74], tedizolid [74], and contezolid [74]) were imported and utilised to identify structural regions of the ligands crucial for bioactivity with the help of the Flare field template [6]. After the conformation hunt, the 2D similarity, shape similarity, and field similarity scores corresponding to the generated template were found to be 0.664, 0.609, and 0.720, respectively. Figure S7 shows the resulting pharmacophore model, displaying important features: positive electrostatic potential (red), negative electrostatic potential (cyan), hydrophobicity (tan), and van der Waals descriptors (yellow). Two well-distinct and separated regions can be identified in the native ligands: a positive electrostatic region and a negative electrostatic region. The top-performing derivatives are displayed for comparison. The orientation of the positive and negative electrostatic regions is consistent amongst all the derivatives, with variations between the width and segmentation of the parts.

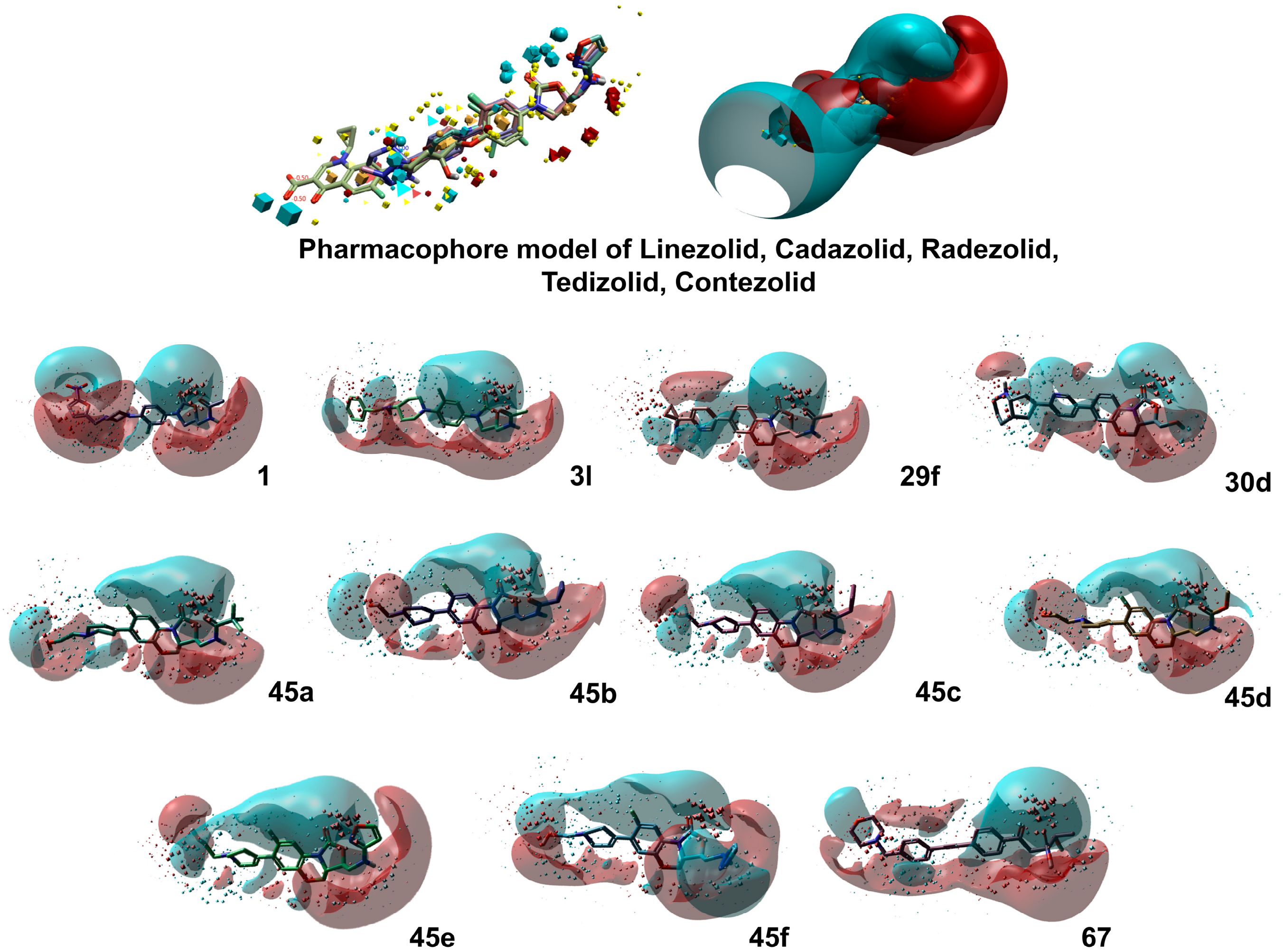

Figure S7. 3D electrostatic–hydrophobic and shape properties. The pharmacophore model generated from linezolid, cadazolid, radezolid, tedizolid, and contezolid presents two distinct and

separated regions: a positive electrostatic potential region in red, and a negative electrostatic potential region, in cyan. The Flare (v.3) module of Cresset software was used to visualise the figure [77].

In addition to the ligand, electrostatics of the binding site can show the complementarity to the ligand and enable the prioritisation of new designs. The electrostatic potential measures the strength of the nearby charges, nuclei, and electrons at a particular position [78]. The positive electrostatic potential is associated with the repulsion of the proton by the atomic nuclei in regions with low electron density and when the nuclear charge is incompletely shielded. At the same time, negative electrostatic potential corresponds to an attraction of the proton by the concentrated electron density in the molecules (from lone pairs and pi-bonds) [79]. Figure S7 demonstrates strong positive and negative electrostatic potential surrounding the varying tail groups attached to the oxazolidinone rings. The more significant areas of cyan and red colour indicate a higher value of negative and positive electrostatics, respectively, leading to potentially higher activity.

When used in conjunction, docking and pharmacophore can complement each other in revealing critical structural features and could be helpful for the development of highly selective and potency of potential drug molecules. An example is the pharmacophore filtering method in which post-processing docking reduces poses and molecules not chemically compatible with the binding site. This includes poses that do not fill the site or leave unpaired buried hydrogen bond donors or acceptors [80]. This method could be viewed as implementing a fundamental principle of structure-based drug design; ligands that bind well must be chemically complementary to their receptors.

Supplementary Method 1. Pharmacophore and Field Template Generation

We performed pharmacophore modelling through a field-based approach using the align molecule wizard in the Flare module of Cresset software [6]. For template generation, the maximum number of conformations was set as 100, conformation hunt and templating calculation methods were set as "Normal" gradient cut-off for conformer minimisation kept at 0.100 kcal/mol/A, and the other parameters were set at default settings. The native ligands of each crystal structure selected earlier were imported from their crystal structure, except for 3CPW, 3DLL, 6DDG, and 6WRU, due to program constraints and high similarity of ligands to other crystal structures (e.g., Isomers). These imported ligands were used as the reference molecules to allow the original positioning of each ligand in its native crystal structure. The template from the FieldTemplater was transferred to Flare and utilised to align the training and test set while building the 3D-QSAR (Quantitative structure-activity relationship) model. For building the 3D-QSAR models, parameters for the conformation hunt were set like that of FieldTemplater. The "very accurate and slow" option was selected for alignment, while the rest of the options remained at the default settings.
